# Association between Serum 25-Hydroxy Vitamin D Levels and the Prevalence of Adult-Onset Asthma

**DOI:** 10.3390/ijerph15061103

**Published:** 2018-05-29

**Authors:** Mark P. C. Cherrie, Christophe Sarran, Nicholas J. Osborne

**Affiliations:** 1 Centre for Research on Environment, Society and Health, Institute of Geography, Drummond Street, Edinburgh EH8 9XP, UK; mark.cherrie@ed.ac.uk; 2 European Centre for Environment and Human Health, University of Exeter Medical School, Truro TR1 3HD, UK; 3 Met Office, FitzRoy Road, Exeter, Devon EX1 3PB, UK; christophe.sarran@metoffice.gov.uk; 4 School of Public Health and Community Medicine, University of New South Wales, Sydney 2052, Australia

**Keywords:** asthma, adulthood, vitamin D, atopy, factor analysis

## Abstract

The major circulating metabolite of vitamin D (25(OH)D) has been implicated in the pathogenesis for atopic dermatitis, asthma and other allergic diseases due to downstream immunomodulatory effects. However, a consistent association between 25(OH)D and asthma during adulthood has yet to be found in observational studies. We aimed to test the association between 25(OH)D and asthma during adulthood and hypothesised that this association would be stronger in non-atopic participants. Using information collected on the participants of the 1958 birth cohort, we developed a novel measure of atopic status using total and specific IgE values and reported history of eczema and allergic rhinitis. We designed a nested case-control analysis, stratified by atopic status, and using logistic regression models investigated the association between 25(OH)D measured at age 46 years with the prevalence of asthma and wheezy bronchitis at age 50 years, excluding participants who reported ever having asthma or wheezy bronchitis before the age of 42. In the fully adjusted models, a 10 nmol/L increase in serum 25(OH)D prevalence had a significant association with asthma (aOR 0.94; 95% CI 0.88–1.00). There was some evidence of an atopic dependent trend in the association between 25(OH)D levels and asthma. Further analytical work on the operationalisation of atopy status would prove useful to uncover whether there is a role for 25(OH)D and other risk factors for asthma.

## 1. Introduction

It is estimated that approximately 18% of the adult population in the UK have had asthma diagnosed by a physician or received medication to treat symptoms [1]. Genetic variants found in chromosome 17q21, female sex hormones, obesity, stress, irritant exposure and environmental pollutants have been implicated in the pathogenesis of adult asthma [2]. Previous studies are limited by the lack of investigation of specific asthma variants or endotypes (i.e., a categorised subtype of adulthood asthma based on pathophysiological signature) [3]. Atopy is regarded as a defining characteristic of the ‘allergic asthma’ endotype [3].

A pooled analysis of seven studies found that 37% (weighted mean) of adulthood asthmatics have their symptoms attributable to SPT defined atopy [4]. This was lower for adult-onset asthma; 21% (95% CI, 8–29) of 19–44 year olds had their condition attributable to atopy [5].

25-Hydroxy vitamin D (25(OH)D) is a pre-hormone, which is converted in the kidney by 1-α-hydroxylase to the active form, 1,25(OH)_2_D [6]. The traditional role of 1,25(OH)_2_D is to ensure calcium homeostasis, although 1,25(OH)_2_D has been shown to have produce immunomodulatory effects by regulating gene expression, due to presence of the vitamin D receptor (VDR) in T cells, B cells, neutrophils, macrophages, and dendritic cells [7]. This may explain significant associations between low 25(OH)D levels and seasonal respiratory infections [8]. 1,25(OH)_2_D has been shown to stimulate Th2-type cytokine production and airway hyperresponsiveness, the suppression of these responses by upregulated Treg cells may provide an explanation to possible beneficial effects on asthma outcomes [9].

In the United Kingdom, vitamin D deficiency has been defined as below or equal to 25 nmol/L, due to the manifestation of rickets and osteomalacia [10]. Based on all the available evidence, recent statements from the National Osteoporosis Society and the Institute of Medicine, recommend sufficient levels as over 50 nmol/L with bone health outcomes as the main endpoint [11]. Optimal levels have been subject to debate, especially with regards to extraskeletal outcomes, a review of 25(OH)D levels for a range of health outcomes determined that 25(OH)D levels over 75 nmol/L was optimum [12]. Therefore, cut-off points of 50 and 75 nmol/L in relation to asthma outcomes are justified [13].

Whilst a large study of Israeli adults found that there was no association between 25(OH)D levels and asthma prevalence [14], the study was not designed to consider the modifying effect of the atopic status of the participants. Given that vitamin D may exert different effects on asthma between atopic and non-atopic individuals, as shown in a recent randomised control trial [15], we hypothesised that the prevalence of asthma in adulthood may be associated with vitamin D levels, but that this association was dependent on atopic status. We firstly aimed to determine whether there was a significant interaction between 25(OH)D and atopic status in the relationship with asthma in adulthood. Next we aimed to investigate whether 25(OH)D continuously and in status categories was associated with adulthood asthma after stratification by atopic status.

## 2. Materials and Methods

### 2.1. Subjects and Sample

**Figure 1:**
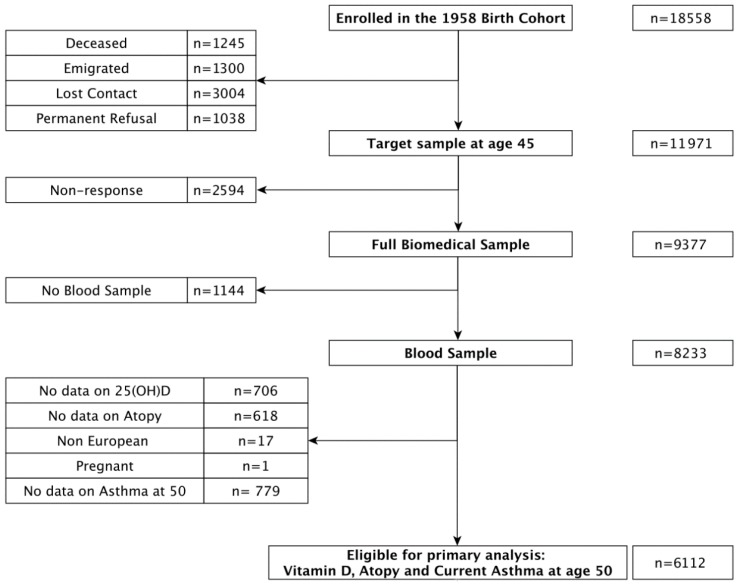
Eligible sample from those that enrolled in the 1958 National Child Development Study.

The 1958 National Child Development Study is Britain-wide sample of residents born during one week in March 1958. A concise cohort profile has been published elsewhere [16]. Briefly, the cohort has been followed up at eight sweeps at age 7, 11, 16, 20, 23, 33, 42, 46 and 50 years. At age 44/45 years, 11,971 cohort participants were still alive, living in Britain and willing to participate were contacted between 2002 and 2004 and invited to be a part of a biomedical survey. The 9377 participants that responded to the invitation were representative of the surviving cohort [17]. Of the 9377, 8233 had blood samples taken successfully. For the current analysis we excluded those with missing data on 25(OH)D levels (*n* = 706), atopy (*n* = 618), and asthma at age 50 years (*n* = 779) and a further 17 cohort members (CM’s) with non-European birth and 1 woman who was pregnant during blood measurement. 6112 participants were eligible for the analysis with asthma prevalence as the outcome (Figure 1).

### 2.2. Biomedical Data at Age 44/45

A team of 122 nurses who were trained to the same experimental protocol, collected venous blood samples and took anthropometric measurements from September 2002 to March 2004, when the participants were age 44/45 years. Total and specific IgE (if the participant recorded higher than the median of 30 kU/L of total IgE) were assayed using the HYTEC Specific IgE Enzyme Immunoassay (EIA). Specific IgE consisted of sensitisation to cat dander, house dust mite and mixed grasses allergen. 25(OH)D levels were assayed using ELISA (IDS OCTEIA Elisa; IDS, Bolton, UK) on the analyzer (BEP 2000; Dade-Behring, Marburg, Germany) [18]. This measurement has been standardised by the mean of over 100 laboratories participating in DEQAS (Vitamin D External Quality Assessment Scheme), which ensured analytical reliability [18]. A tension tape was used to measure waist circumference at the midpoint, defined as between the lower ribs and iliac crest, in centimetres. BMI was defined as weight divided by height squared.

### 2.3. Self-Reported Data at Age 42

The participants were interviewed and responded to questions on aspects of their health and lifestyle at age 42. Eczema history was defined as a positive response to the question, “Cohort member ever had eczema?” The same question was used to assess the presence of “allergic rhinitis, persistent runny nose”. Smoking status was assessed and categorised into current smoker, ex/occasional smoker and never smoked. Social class based on occupation (formerly Registrar General’s Social Class) and categorised as: “Professional (I)”, “Managerial-technical (II)”, “Non-manual (III)”, “Manual (III)”, “Partly skilled (IV)” and “Unskilled (V)” and available for both age 42 and birth (based on father’s occupation). Physical activity was defined in response to the question, “How often CM takes part in any exercise activity?”, with categories of “Every day”, “4–5 days a week”, “2–3 days a week”, “Once a week”, “2–3 times a month” and “Less Often”.

### 2.4. Self-Reported Data at Age 44/45

The participants were interviewed and responded to questions on aspects of their occupation and lifestyle at age 44/45. Occupational dust exposure was assessed with the question: “Have you ever worked in a place with a lot of dust?” The responses were categorised as “No, never”, “Yes, in the last 2 years” and “Yes, more than 2 years ago”. Ordinal responses were also recorded in response to questions on indoor activity (“How much time on average did you spend watching TV last year?”), outdoor activity (“How long per day do/did you usually spend outdoors in during daylight?”) and oily fish consumption (“how often do you eat other fish (oily fish) such as salmon, trout, mackerel, sardines, fresh tuna?”). Vitamin D supplementation in the last month was defined as supplements containing vitamin in the previous month (“what do these supplements contain?”). The dose, timing and form (D_2_ or D_3_) were unknown. Region of residence was defined as South (South East, South West), Greater London, Middle England and Wales (East Anglia, Midlands, and Wales), Northern England (North, North West, and Yorkshire and the Humber), and Scotland.

### 2.5. Self-Reported Asthma Data

Asthma prevalence was defined by whether the cohort member suffers asthma or wheezy bronchitis at age 50. We excluded participants who had previously responded positively to the question of ever having asthma or wheezy bronchitis before the age of 42.

### 2.6. Operationalisation of 25(OH)D Level and Status

The blood samples were taken at varying months over the course of two years, with a slightly higher frequency in autumn (38% of the sample). Given that 25(OH)D levels display a considerable seasonal variation, each model was adjusted by the season of blood measurement. Vitamin D status was analysed continuously by 10 nmol/L but also categorised as insufficient (<50 nmol/L), sufficient (50–75 nmol/L) and optimal (>75 nmol/L). The insufficient category was used as the reference category in the regression models.

### 2.7. Operationalisation of Atopy Score and Categories

Measurements of total IgE, specific IgE (HDM, cat and grass) were used alongside responses relating to a history of eczema and allergic rhinitis to predict an atopy score using principal components analysis. Factors with eigenvalues above 1 were selected and rotated using varimax rotation. Factor loadings were as follows total IgE (0.65), specific IgE to cat dander (0.31), specific IgE to grass (0.45), specific IgE to house dust mite (0.49), history of eczema (0.15) and history of asthma (0.15). The atopy score was split into tertiles corresponding to low, moderate and high categories. A sensitivity analysis was undertaken with total IgE, dichotomised using the threshold of 160 kU/L and specific IgE measurements to either house dust mite, cat or grass, dichotomised by a threshold of 0.35 kU/L.

### 2.8. Statistical Analysis

Univariate descriptive statistics were generated for each of the variables with mean and standard deviations presented for continuous variables and numbers with percentages for categorical variables. For 25(OH)D geometric means and 95% confidence intervals were presented with adjustment for sex and season. The interaction between 25(OH)D levels and atopic score was tested by the likelihood ratio test, and an investigation of the linearity of the relationship between continuous variables was tested using the user-written “mfpigen” package in Stata. Variables with observations answered with non-response (e.g., “not answered”) or lack of clarity (e.g., “don’t know”) were coded as missing. Multiple imputation of covariate missing data was achieved by chained equations using Stata’s ‘MI’ command on the assumption that data was missing at random. The results presented are derived from the multiple imputed datasets. Three nested models were developed: model 1 was adjusted for sex and season, model 2 was additionally adjusted for smoking status, TV and PC time, physical activity, outdoor activity, oily fish consumption, vitamin D supplementation, region of residence, occupational socioeconomic position (SEP) at birth (father’s occupation) and adulthood, model 3 was additionally adjusted for adiposity measures (BMI, BMI squared, waist circumference, waist circumference squared). We investigated the association of 25(OH)D levels in all participants, then by atopic participants in a stratified analysis. Odds ratio and 95% confidence intervals are presented, with significance level set at *p* < 0.05. The analysis was performed using Stata 12.1 (Stata Corp., College Station, TX, USA).

## 3. Results

### 3.1. Descriptive Statistics

There was no clear gradient in the level of 25(OH)D by SEP (birth or in adulthood) or occupational dust exposure. Participants who were male, consumed less alcohol/more cigarettes, lived further North, spent less time outdoors in summer and more time watching TV, did not take vitamin D supplements or ate oily fish regularly, and were obese, had lower levels of 25(OH)D. The relationship between demographic, behavioural and occupational variables with atopy categories was less clear but those with the lowest atopy score tended to be males, non-smokers, have higher alcohol consumption, no previous occupational dust exposure and were not obese. Participants reporting adulthood asthma were more likely to be males, in a lower SEP (especially in adulthood but also in birth), with higher consumption of cigarettes, more time spent watching TV, lower consumption of oily fish and were recorded as being obese (Table 1).

### 3.2. 25(OH)D and Atopy Interaction

**Figure 2:**
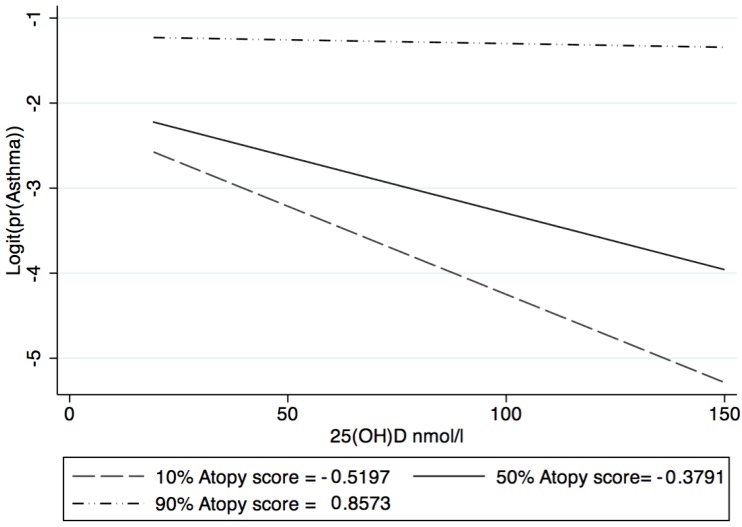
Association between 25(OH)D and asthma by atopy centile.

The best fit for the interaction between 25(OH)D levels and atopic score was the linear form (*p* < 0.01). The association between 25(OH)D levels and odds of asthma, is presented for the 10th, 50th and 90th atopy score percentile in Figure 2.

At the 90th percentile of atopy score (highly atopic) there was a higher odds of asthma but no difference by the level of 25(OH)D. At the 50th percentile (moderately atopic) there was a modest and gradual decline in the odds of asthma with an increase in 25(OH)D level. Finally, at the 10th percentile (low atopic) there was a steep decline in the odds of asthma with increasing 25(OH)D.

### 3.3. Association between 25(OH)D and Asthma, by Atopy Category

Asthma was shown to inversely associate with 25(OH)D levels, for each 10 nmol/L increase in 25(OH)D there was a 6% (0–12%) reduction in the odds of asthma, when compared to those with 25(OH)D levels above 50 nmol/L (Table 2). This association was stronger, but non-significant in the low atopic participants (aOR 0.87, 95%CI 0.74–1.02), with a declining effect size for those classified as moderate 0.92 (0.82–1.04) and highly atopic (aOR 0.98, 95%CI 0.91–1.05). Although a gradient of association was found between 25(OH)D status categories (i.e., those in the optimal category had the lowest probability of asthma with the effect size varied depending on atopic status), these too were insignificant. In the sensitivity analysis with simpler definitions of atopy, there was the same inference (Tables S1 and S2). For example using specific IgE categories, those defined as low atopic had an odds ratio of 0.91 (0.86–0.98), per 10 nmol/L increase in 25(OH)D. Similar to the main results, the odds of asthma was not statistically different by vitamin D status category in the sensitivity analysis.

## 4. Discussion

### 4.1. Main Findings

We found a significant interaction between 25(OH)D levels and atopy when investigating the relationship with asthma in adulthood, in unadjusted models. Although, 25(OH)D was associated with asthma in all participants, we found that those with the lowest atopy scores, as defined by biomarkers and history of allergic disease had some of the strongest associations, but that these failed to be statistically significant after adjustment for adiposity. There was also some evidence that these associations were sensitive to the classification of atopy and whether 25(OH)D was used continuously or in status categories. These results have important implications for future work.

### 4.2. Strengths and Limitations

This study has utilised cohort data from the large 1958 National Child Development Study. The analysis was undertaken in subsamples of participants with complete data on the 25(OH)D levels, atopy and asthma. In the adjusted models, imputation of missing covariate variables was undertaken. The subsamples are not representative of the British population in terms of age and ethnicity of the current UK population, although this gave us power to investigate the relationship in middle-aged white Britons. The stratification by atopic status was undertaken with respect to guidelines proposed by Pekkanen et al. [19]. The novel use of an exploratory factor analysis to determine atopy status rather than solely IgE or SPT is based on the acknowledgement that atopy is a multifactorial syndrome associated with allergic sensitisation and history of disease. Models were comprehensively adjusted for by risk factors for adulthood asthma, including measures that have not been frequently used in other past analyses of the cohort i.e., occupational dust exposure. This allowed for a robust presentation of how the relationship between 25(OH)D levels and asthma may be modified by atopic status, considering the importance of occupational stressors in adult asthma.

There is the possibility that due to using subsets of the biomedical survey participants, the analysis samples may not be representative of the surviving cohort in terms of unselected measured variables and unknown unmeasured variables. By subsetting the data, there were lower numbers of atopic asthmatics, which may partly explain the lack of associations for this subgroup. There is also the potential for misclassification of Chronic Obstructive Pulmonary Disease (COPD) as asthma in adulthood is an issue that has been discussed elsewhere [20], clustering by pathophysiological parameters (e.g., FOXP3 (Treg)) should be adopted, when this data is available, to determine non-atopic and late-onset asthma in future analyses [21]. Outcome misclassification was considered in the study design (i.e., age of cohort) and analysis (i.e., adjustment for smoking status) but it is acknowledged that COPD in the outcome may persist and partly explain associations presented. We assume that a measurement of vitamin D at a single time point in adulthood can act as a marker for long term exposure level and that 25(OH)D levels is suitable marker for bioavailable vitamin D, both of which have been contested in the literature [22]. While use of vitamin D supplements was recorded we did not know the amount or timing of the supplements, but it still allowed us to adjust for vitamin D supplements in our models. We did not take into account any medication that may influence vitamin D levels, however a descriptive analysis revealed that there was some support for an association between vitamin D status and medication for cardiovascular, Central Nervous System, Obs/gynae/UTI and musculo-skeletal/Joint outcomes (results not shown). Furthermore, more detailed information on medication use (e.g., Glucocorticoids) could further distinguish between asthma subtypes in future analyses.

### 4.3. Relation to Previous Work

The main results are in line with the results from a recent study using SPT defined atopy status [23]. The authors found a lower prevalence of non-atopic asthmatic participants closest to the equator or with the highest exposure to current/cumulative UV-B (i.e., higher 25(OH)D level) [23]. Similarly, in a Norwegian cohort, non-atopic males classified by their absence of allergic rhinitis with vitamin D levels below 50 nmol/L had a doubling of risk of incident asthma (adjusted odds ratio of 2.32 (95% CI: 1.06, 5.10) [13]. In the same cohort, men with asthma, no history of allergic rhinitis and serum 25(OH)D levels below 50 nmol/L had a lower level of FEV1/FVC ratio (β = −8.60; 95% CI: −16.95% to −0.25%) [24]. It was shown that there are differences in the effect of risk factors for atopic and non-atopic asthma in childhood [25]. A differential effect of vitamin D on asthma by atopic status may explain why associations between latitude and asthma have not been found in childhood, when atopic asthma is more prevalent, despite relationships with food allergy and eczema being significant in the same cohort [26]. Adult-onset asthma is predominantly non-atopic and characterised by severe symptoms including a fast decline in lung function [2], which have been associated with 25(OH)D levels previously [27]. In the same way as there is a ‘window of opportunity’ to alter the in utero environment to prevent childhood atopic asthma [28], adulthood non-atopic asthma may have a window much closer to diagnosis i.e., early adulthood, although whether this is a critical period requires investigation in future life-course analyses. Several risk factors including occupational exposures, smoking, female sex hormones, obesity, stressful life events, aspirin/paracetamol use, upper airway disease, and respiratory infections have been implicated [2]. In previous analyses of the 1958 National Child Development Study, 16% of adult onset asthma was estimated to be due to occupational exposures [29]. Undertaking a similar type of analysis as we have presented in a larger cohort, e.g., UK Biobank, would allow for investigation of multiple risk factors together. There may be more scope for environmental intervention on the course of non-atopic asthma, given the predominance of non-genetic risk factors. From the results of this study it would seem that raising 25(OH)D levels would be more appropriate rather than to attempt to attain a certain status level. Given that the relationship between adiposity and circulating 25(OH)D is well established [30] and there is evidence that weight loss improves 25(OH)D levels [31], it may be appropriate to recommend weight loss in adulthood (with vitamin D supplementation in patients with the lowest 25(OH)D levels) to lower the risk of non-atopic asthma in adulthood, although this hypothesis should be tested in a prospective, longitudinal study.

## 5. Conclusions

Examination of asthma as distinct endotypes such as atopic versus non-atopic asthma may reveal further risk factors and provide evidence of different pathological pathways. We found differing results by different definitions of atopic status, with the most complex showing no significant associations after adjustment for adiposity measures. A standardised methodology for the investigation of atopic status should be developed as current heterogeneity in study measures limit meta-analyses and this might highlight certain risk factors for adulthood asthma currently masked. Future studies should also investigate the interaction between sets of risk factors, such as 25(OH)D and weight gain/loss, to understand whether a complex intervention would be effective.

## Figures and Tables

**Table 1:** Selected characteristics of participants.

Selected Characteristic at Age 45	25(OH)D (nmol/L) at Age 45 *	Atopy **			Adulthood Asthma or Wheezy Bronchitis at Age 50 ***	
		Q1: Low	Q2: Moderate	Q3: High	No	Yes
Sex						
Male	50.1 (49.5–50.7)	899 (37.6)	1030 (33.0)	915 (29.4)	2598 (90.6)	267 (9.3)
Female	48.6 (48.0–49.2)	1172 (30.4)	974 (32.7)	1110 (37.2)	2564 (94.1)	161 (5.9)
Socioeconomic Position at 42						
Professional/Managerial-Technical	49.7 (49.0–50.3)	842 (32.9)	824 (32.2)	893 (34.9)	2200 (93.2)	160 (6.7)
Skilled (non-manual)	49.1 (48.1–50.2)	471 (36.2)	441 (33.9)	389 (29.9)	1096 (91.8)	98 (8.2)
Skilled (manual)	50.0 (48.8–51.3)	373 (32.4)	379 (33.0)	398 (34.6)	973 (92.8)	75 (7.2)
Unskilled and others	47.9 (46.8–49.0)	320 (35.1)	312 (34.2)	280 (30.7)	742 (90.1)	82 (10.0)
NA	178 (3)					
Socioeconomic Position at birth						
Professional/Managerial-Technical	49.8 (48.9–50.7)	393 (32.7)	395 (32.9)	413 (34.4)	1029 (93.1)	76 (6.9)
Skilled (non-manual)	51.4 (50.0–52.7)	212 (34.5)	199 (32.7)	204 (33.2)	534 (92.4)	44 (7.6)
Skilled (manual)	49.5 (48.9–50.1)	1019 (34.4)	968 (32.7)	978 (33.0)	2507 (92.5)	202 (7.5)
Unskilled and others	47.9 (47.0–48.8)	409 (34.8)	398 (33.9)	1174 (31.3)	974 (91.0)	96 (8.9)
NA	145 (2)					
Smoking status						
Never	50.1 (49.5–50.7)	1026 (36.0)	879 (30.8)	946 (33.2)	2446 (92.5)	199 (7.5)
Ex-Smoker	50.8 (50.0–51.6)	551 (34.6)	530 (33.3)	511 (32.1)	1339 (92.1)	115 (7.9)
1–19 a day	48.4 (47.3–49.5)	285 (29.1)	358 (36.5)	337 (34.4)	828 (92.8)	64 (7.2)
≥20 a day	44.0 (42.8–45.2)	206 (30.8)	235 (32.9)	227 (34.0)	541 (91.7)	49 (8.3)
NA	9 (0)					
Alcohol consumption						
Not in the last 12 months	44.3 (42.7–45.8)	131 (36.8)	122 (34.3)	103 (28.9)	293 (89.1)	36 (10.9)
Once a month or less	45.3 (44.3–46.4)	314 (38.1)	246 (30.0)	264 (32.0)	683 (89.9)	77 (10.1)
2–4 times a month	49.6 (48.8–50.5)	502 (38.0)	420 (31.8)	400 (30.3)	1135 (93.5)	79 (6.5)
2–3 times a week	51.1 (50.4–51.8)	630 (31.5)	678 (33.9)	690 (34.5)	1687 (92.1)	145 (7.9)
Over 4 times a week	50.3 (49.5–51.2)	491 (31.0)	533 (33.6)	561 (35.4)	1351 (93.8)	90 (6.3)
NA	15 (0)					
Region						
Scotland	44.0 (42.8–45.2)	220 (36.1)	192 (31.5)	197 (32.4)	513 (91.6)	47 (8.4)
Northern England	49.7 (48.9–50.5)	534 (33.3)	549 (34.9)	500 (31.8)	1337(92.65)	106 (7.4)
Middle England and Wales	49.1 (48.3–50.0)	443 (33.4)	423 (32)	461 (34.7)	1101 (91.9)	97 (8.1)
Greater London	47.2 (45.7–48.8)	795 (35.0)	702 (32.4)	709 (32.7)	353 (92.4)	29 (7.6)
Southern England	51.0 (50.3–51.7)	125 (29.7)	138 (32.8)	158 (37.5)	1858 (92.6)	149 (7.4)
NA	-					
TV time (hours a day)						
<1	51.1 (50.0–52.3)	280 (34.4)	289 (35.6)	244 (30.0)	698 (93.3)	50 (6.7)
1 to <3	50.0 (49.5–50.5)	1335 (34.5)	1251 (32.3)	1282 (33.1)	3290 (92.6)	265 (7.5)
≥3	46.7 (45.8–47.5)	1251 (32.3)	421 (32.3)	463 (35.5)	1082 (91.2)	104 (8.8)
NA	115 (2)					
Time Spent outdoors in summer (hours/day)						
<1	43.9 (42.7–45.3)	182 (37.9)	147 (30.6)	151 (31.5)	404 (92.0)	35 (8.0)
1 to <3	48.2 (47.4–49.1)	430 (32.6)	434 (32.9)	455 (34.5)	1135 (92.5)	92 (7.5)
≥3	50.5 (50.0–51.1)	1308 (34.0)	1266 (32.9)	1274 (33.1)	3243 (92.5)	264 (7.5)
NA	453 (7)					
Vitamin D supplementation						
No	48.4 (48.0–48.9)	1708 (34.1)	1634 (32.7)	1662 (33.2)	4233 (92.4)	346 (7.6)
Yes	55.9 (54.6–57.2)	223 (33.0)	228 (33.8)	224 (33.2)	582 (92.5)	47 (7.5)
NA	421 (7)					
Oily fish consumption						
Never	45.9 (44.8–47.0)	268 (35.6)	253 (33.6)	232 (30.8)	620 (90.3)	67 (9.8)
Less than weekly	49.4 (48.8–49.9)	1137 (33.6)	1129 (33.3)	1120 (33.1)	2888 (93.0)	219 (7.1)
Weekly	51.1 (50.3–51.9)	636 (34.4)	585 (31.7)	626 (33.4)	1565 (92.4)	129 (7.6)
NA	114 (2)					
Occupational Dust Exposure						
No, never	49.6 (49.0–50.1)	1382 (35.6)	1264 (32.5)	1242 (31.9)	3347 (93.4)	236 (6.6)
Yes, in the last two years	49.6 (48.1–51.1)	244 (32.6)	255 (34.1)	250 (33.4)	634 (92.3)	53 (7.7)
Yes, more than two years ago	47.1 (46.0–48.3)	329 (30.6)	348 (32.4)	397 (37.0)	866 (89.8)	98 (10.2)
NA	389 (6)					
Obesity (BMI > 30)						
No	51.1 (50.6–51.5)	1596 (34.9)	1501 (32.8)	1483 (32.4)	3931 (92.9)	299 (7.1)
Yes	44.4 (43.7–45.1)	475 (31.3)	503 (33.1)	542 (35.7)	1231 (90.5)	129 (9.5)
NA	-					

* Geometric mean adjusted for sex and season; ** Atopy score was determined by exploratory factor analysis using concentrations of total IgE, specific IgE (cat, grass, HDM) and responses to questions relating to history of eczema and current allergic rhinitis; *** Those who responded positively to having asthma before 42 were excluded.

**Table 2:** Association between 25(OH)D levels and asthma at age 50, by atopic status category.

Atopy Category	Vitamin D Status			25(OH)D Level
	Insufficient (<50 nmol/L)	Sufficient (50–75 nmol/L)	Optimal (>75 nmol/L)	Per 10 nmol/L Increase
All categories				
Model 1 ¶	Ref.	0.84 (0.67–1.05)	0.69 (0.48–0.98) *	0.91 (0.86–0.96) **
Model 2 #	Ref.	0.88 (0.70–1.10)	0.71 (0.49–1.02)	0.92 (0.86–0.97) **
Model 3 ¥	Ref.	0.94 (0.74–1.19)	0.77 (0.52–1.12)	0.94 (0.88–1.00) *
Low atopic				
Model 1 ¶	Ref.	0.70 (0.43–1.13)	0.43 (0.18–1.05)	0.79 (0.69–0.92) **
Model 2 #	Ref.	0.73 (0.44–1.22)	0.46 (0.18–1.12)	0.80 (0.69–0.93) **
Model 3 ¥	Ref.	0.89 (0.52–1.52)	0.62 (0.25–1.58)	0.87 (0.74–1.02)
Moderate atopic				
Model 1 ¶	Ref.	0.90 (0.61–1.32)	0.50 (0.25–1.00) *	0.89 (0.80–0.99) *
Model 2 #	Ref.	0.98 (0.66–1.47)	0.50 (0.24–1.05)	0.91 (0.82–1.02)
Model 3 ¥	Ref.	1.08 (0.71–1.63)	0.45 (0.20–1.04)	0.92 (0.82–1.04)
High atopic				
Model 1 ¶	Ref.	0.87 (0.67–1.13)	0.94 (0.63–1.39)	0.96 (0.90–1.02)
Model 2 #	Ref.	0.89 (0.68–1.17)	0.96 (0.64–1.44)	0.96 (0.90–1.03)
Model 3 ¥	Ref.	0.92 (0.69–1.21)	1.02 (0.68–1.55)	0.98 (0.91–1.05)

¶ Adjusted for sex and season; # additionally adjusted for smoking status at age 42, TV and PC time, physical activity at age 42, outdoor activity at age 46, oily fish consumption, vitamin D supplementation at age 46, region of residence at age 46, occupational social class at birth (father’s occupation) and at age 46; ¥ additionally adjusted BMI, BMI squared, waist circumference, waist circumference squared. * *p* < 0.05, ** *p* < 0.01.

## Supplementary Materials

The following are available online at http://www.mdpi.com/1660-4601/15/6/1103/s1, Table S1: Sensitivity Analysis—The association between 25(OH)D levels and asthma prevalence at age 50, by specific IgE category, Table S2: Sensitivity Analysis-The association between 25(OH)D levels and asthma prevalence at age 50, by total IgE category.
