# Supplementary material for: Association between Serum 25-Hydroxy Vitamin D Levels and the Prevalence of Adult-Onset Asthma
Source: Int J Environ Res Public Health. 2018 May 29;15(6):1103. doi: 10.3390/ijerph15061103 (PMC6025639; doi:10.3390/ijerph15061103)
Supplement: Supplementary file 1 [file ijerph-15-01103-s001.pdf]

International Journal of *Environmental Research and Public Health*

MDPI

Supplementary Materials: Association Between Serum 25-Hydroxy Vitamin D Levels and the Prevalence of Adult-Onset Asthma

Mark P. C. Cherrie [1,2], Christophe Sarran [3] and Nicholas J. Osborne [2,4,*]

[1] Centre for Research on Environment, Society and Health, Institute of Geography, Drummond Street, Edinburgh EH8 9XP, UK; mark.cherrie@ed.ac.uk

[2] European Centre for Environment and Human Health, University of Exeter Medical School, Truro, TR1 3HD, UK

[3] Met Office, FitzRoy Road, Exeter, Devon EX1 3PB, UK; christophe.sarran@metoffice.gov.uk

[4] School of Public Health and Community Medicine, University of New South Wales, Sydney Australia 2052, Australia

* Correspondence: n.osbone@unsw.edu.au; Tel.: +61-293851929 or +61-431854846

Table S1. Sensitivity Analysis- The association between 25(OH)D levels and asthma prevalence at age 50, by specific IgE category.

	Vitamin D status			25(OH)D level
	**Insufficient (<50 nmol/L)**	**Sufficient (50–75 nmol/L)**	**Optimal (>75 nmol/L)**	**Per 10 nmol/L increase**
Low Specific IgE (<0.35 kU/L)				
Model 1 [¶]	Ref	0.81 (0.65–1.01)	0.59 (0.41–0.85)**	0.88 (0.83–0.93)**
Model 2 [#]	Ref	0.83 (0.66–1.04)	0.60 (0.42–0.89)**	0.88 (0.83–0.94)**
Model 3 [¥]	Ref	0.92 (0.73–1.16)	0.68 (0.46–1.01)	0.91 (0.86–0.98)*
High Specific IgE (>0.35 kU/L)				
Model 1 [¶]	Ref	0.96 (0.63–1.45)	1.24 (0.73–1.03)	1.01 (0.91–1.12)
Model 2 [#]	Ref	0.97 (0.63–1.51)	1.28 (0.67–2.44)	1.03 (0.93–1.15)
Model 3 [¥]	Ref	0.94 (0.60–1.47)	1.28 (0.66–2.48)	1.03 (0.92–1.16)
Adjusted for sex and season				

[#] additionally adjusted for smoking status at age 42, TV and PC time, physical activity at age 42, outdoor activity at age 46, oily fish consumption, vitamin D supplementation at age 46, region of residence at age 46, occupational social class at birth (father's occupation) and at age 46; [¥] additionally adjusted BMI, BMI squared, waist circumference, waist circumference squared. * p <0.05, ** p <0.01.

Table S2. Sensitivity Analysis-The association between 25(OH)D levels and asthma prevalence at age 50, by total IgE category.

	Vitamin D status			25(OH)D level
	**Insufficient (<50 nmol/L)**	**Sufficient (50–75 nmol/L)**	**Optimal (>75 nmol/L)**	**Per 10 nmol/L increase**
Low total IgE (<160kU/L)				
Model 1 [¶]	Ref	0.79 (0.60–1.03)	0.55 (0.35–0.87)*	0.86 (0.80–0.93)**
Model 2 [#]	Ref	0.83 (0.63–1.10)	0.58 (0.36–0.93)*	0.88 (0.82–0.95)**
Model 3 [¥]	Ref	0.92 (0.69–1.23)	0.64 (0.39–1.05)	0.91 (0.84–0.99)*
High total IgE (>160kU/L)				
Model 1 [¶]	Ref	0.88 (0.67–1.17)	0.91 (0.59–1.40)	0.95 (0.89–1.03)
Model 2 [#]	Ref	0.92 (0.68–1.24)	0.96 (0.61–1.51)	0.97 (0.89–1.04)
Model 3 [¥]	Ref	0.96 (0.71–1.29)	1.03 (0.65–1.64)	0.98 (0.91–1.06)
Adjusted for sex and season				

[#] additionally adjusted for smoking status at age 42, TV and PC time, physical activity at age 42, outdoor activity at age 46, oily fish consumption, vitamin D supplementation at age 46, region of residence at age 46, occupational social class at birth (father's occupation) and at age 46; [¥] additionally adjusted BMI, BMI squared, waist circumference, waist circumference squared. * p <0.05, ** p <0.01.

© 2017 by the authors. Submitted for possible open access publication under the terms and conditions of the Creative Commons Attribution (CC BY) license (http://creativecommons.org/licenses/by/4.0/).
